# Interleukin-17 and matrix metalloprotease-9 expression in the mycetoma granuloma

**DOI:** 10.1371/journal.pntd.0007351

**Published:** 2019-07-11

**Authors:** Emmanuel Edwar Siddig, Ali Mahmoud Mohammed Edris, Sahar Mubarak Bakhiet, Wendy W. J. van de Sande, Ahmed Hassan Fahal

**Affiliations:** 1 The Mycetoma Research Centre, University of Khartoum, Khartoum, Sudan; 2 Faculty of Medical Laboratory Sciences, University of Khartoum, Khartoum, Sudan; 3 ErasmusMC, University Medical Centre Rotterdam, Department of Medical Microbiology and Infectious Diseases, Rotterdam, The Netherlands; 4 Institute for Endemic Diseases, University of Khartoum, Khartoum, Sudan; Faculty of Science, Ain Shams University (ASU), EGYPT

## Abstract

Mycetoma is a persistent, progressive granulomatous inflammatory disease caused either by fungi or by bacteria. Characteristic of this disease is that the causative agents organise themselves in macroscopic structures called grains. These grains are surrounded by a massive inflammatory reaction. The processes leading to this host tissue reaction and the immunophenotypic characteristics of the mycetoma granuloma are not known. Due to the massive immune reaction and the tissue remodeling involved, we hypothesised that the expression levels of interleukin-17 (IL-17) and matrix metalloprotease-9 (MMP-9) in the mycetoma granuloma formation were correlated to the severity of the disease and that this correlation was independent of the causative agent responsible for the granuloma reaction. To determine the expression of IL-17 and MMP-9 in mycetoma lesions, the present study was conducted at the Mycetoma Research Centre, Sudan. Surgical biopsies from 100 patients with confirmed mycetoma were obtained, and IL-17 and MMP-9 expression in the mycetoma granuloma were evaluated immunohistochemically. IL-17 was mainly expressed in Zones I and II, and far less in Zone III. MMP-9 was detected mainly in Zones II and III, and the least expression was in Zone I. MMP-9 was more highly expressed in *Actinomadura pelletierii* and *Streptomyces somaliensis* biopsies compared to *Madurella mycetomatis* biopsies. MMP-9 levels were directly proportional to the levels of IL-17 (*p* = 0.001). The only significant association between MMP9 and the patients’ characteristics was the disease duration (*p*<0.001). There was an insignificant correlation between the IL-17 levels and the patients’ demographic characteristics.

## Author summary

Mycetoma is a progressive and destructive, chronic granulomatous tumourous inflammatory disease, which is caused by a wide range of bacterial and fungal organisms. These causative agents form grains inside the tissue and a large inflammation zone and collagen capsule is found to be surrounding them. How these grains and the surrounding tissue reaction are formed is currently not known. In this study we demonstrated that IL-17A and MMP-9 were expressed in the mycetoma granuloma but at different zones surrounding the grain. The MMP-9 levels were directly proportional to the IL-17A levels. The highest IL-17A expression was found in the largest lesions and the lesions with the longest duration. These lesions are often also associated with a continuous influx of neutrophils and tissue damage and remodeling due to the production of ROS and metalloproteases and this could explain the correlation found between IL-17A and MMP-9 in mycetoma lesions.

## Introduction

Mycetoma is a common neglected disease, endemic in many tropical and subtropical areas [1]. It is a chronic granulomatous inflammatory disease, which usually spreads to involve the skin, deep structures and leads to massive deformities, disabilities and deformities [2]. It usually affects young adults and children, is commonly seen in farmers and workers dealing with the soil [3, 4] and has many negative impacts on patients, families and community [5, 6].

Fifty-Six different microorganisms of both bacterial (actinomycetoma) and fungal origin (eumycetoma), can cause mycetoma [7–9]. In Sudan, the most commonly encountered micro-organisms are the fungus *Madurella mycetomatis* and the bacteria *Streptomyces somaliensis*, *Actinomadura madurae* and *Actinomadura pelletieri*. Clinically, mycetoma presents as a slow growing tumour-like soft tissue mass that gradually increases in size. With time, multiple sinuses develop, and eventually, they drain purulent and seropurulent discharge that contains grains [10, 11]. These grains which are characteristic of mycetoma are of different colours, sizes and consistency depending on the causative organisms [12, 13]. *M*. *mycetomatis* produces black grains, *S*. *somaliensis* forms yellow grains and *A*. *pelletieri* forms red grains.

Although these micro-organisms form grains of different color and consistency, the tissue-reaction surrounding these grains are quite similar. In general, the grains are surrounded by three zones of inflammatory cells. The innermost zone, zone 1, consists mainly of neutrophils [14]. The following zone, zone 2 consists mainly of macrophages and the outermost zone, zone 3, contains lymphocytes and plasma cells [15]. Furthermore, encapsulation by collagen fibers was also demonstrated [16]. Although the inflammation reaction between eumycetoma and actinomycetoma is similar, there are some differences. The number of CD8+ lymphocytes and macrophages was higher in actinomycetoma lesions than in eumycetoma lesions [17]. In order to find some clues about the type of immune reaction needed to establish a mycetoma granuloma, several immunological studies were carried out to address these aspects. Few studies have reported the role of cellular immunity in human [18]. Furthermore, El Hassan and colleagues determined the cytokine profile in the inflammatory reaction surrounding *S*. *somaliensis* lesions [15]. Th2 cytokines interleukin (IL)-4 and IL-10 were found to be mainly expressed while Th1 cytokine interferon γ (INF-γ) was only expressed in 1 out of 3 patients [15], moreover, Mhmoud and her associates determined the association between Th2 cytokine namely IL-10 in the granuloma caused by *M*. *mycetomatis*. Their results showed that IL-10 was expressed in all zones surrounding the grains of *M*. *mycetomatis* [19]. At the time of that study, the authors mainly focused on the Th1 and Th2 response, the Th17 response was not investigated. However, in the following years more insight was obtained in the Th17 response. The Th17 response appeared to play an important role the subcutaneous fungal implantation mycosis chromoblastomycosis [20], and in experimental *Nocardia brasiliensis* mycetoma in mice [21]. Th17 cells are a unique subset of CD4^+^ T-cells and are characterized by production of interleukin-17 (IL-17) [22]. IL-17 is a family of six cytokines known as IL-17A till F [23]. IL-17A coordinates the tissue inflammation through higher expression of pro inflammatory cytokines [24] and activation of epithelial cells and fibroblasts [25, 26]. This in its turn results in the release of interleukin 6 (IL-6), interleukin 8 (IL-8) and tumor necrosis factor alpha (TNFα) in macrophages, fibroblasts, and endothelial cells which ultimately lead to pronounced neutrophil infiltration [27–31]. A phenomenon also seen in mycetoma lesions. IL-17A then activates the neutrophils to produce rapid oxygen species (ROS) in order to destroy the extracellular micro-organism [32]. More IL-17A will also be produced to attract even more neutrophils to the site of infection [32], which results in even a higher production of ROS. Unfortunately ROS is not specific in killing micro-organisms and local tissue damage will also occur. To be able to respond to this tissue damage, matrix metalloproteases (MMPs) will be up-regulated in order to remodel the tissue matrix [33, 34]. IL-17 is known to up-regulate of matrixmetalloprotease-2 (MMP-2) and MMP-9, both responsible for the formation of collagen capsules [35, 36]. Therefore it was not surprising that also in neutrophilic skin conditions such as mucosal leishmaniasis [37] and amicrobial pustulosis of the folds [38], a correlation between IL-17 and MMP-9 expression seemed to be present. Also in mycetoma lesions both MMP-2 and MMP-9 are expressed at the site of infection [16], however to date there is no data indicating whether a Th17 response plays a role in the mycetoma granuloma formation and if there is a correlation between IL-17A expression and MMP expression in mycetoma lesions. To address this question, as a proof of principle, we assessed the IL-17A and MMP-9 expression in the mycetoma granuloma caused by *M*. *mycetomatis*, *S*. *somaliensis* and *A*. *pelletieri* using an immunohistochemical (IHC) appraoch and correlated their expression levels with the clinical characteristics of the patients.

## Materials and methods

This descriptive study was conducted at the Mycetoma Research Centre, Soba University Hospital, University of Khartoum, Khartoum, Sudan. The study included 100 patients with different confirmed types of mycetoma. The diagnosis was confirmed by meticulous clinical examinations, culture of grains and histopathological examination of material obtained by a surgical biopsy. Surgical biopsies from these patients were fixed in 10% formalin for 24 hours, and paraffin blocks were prepared. All sections were stained with haematoxylin and eosin (H&E). The mycetoma causative organisms in the studied patients were *M*. *mycetomatis* (n = 80), *S*. *somaliensis* (n = 12) and *A*. *pelletierii* (n = 8), a representation of the distribution of causative agents normally seen in the Mycetoma Research Centre.

### Collagen fibers detection

Collagen fibers were detected in tissue sections by staining them in Weigert’s iron hematoxylin working solution for 10 minutes. After rinsing in running warm tap water for 10 minutes and washing in distilled water they were stained with scarlet-acid fuchsine solution for 10–15 minutes and washed in distilled water. They were differentiated in phosphomolybdic-phosphotungstic acid solution for 10–15 minutes or until collagen was not red. Sections were transferred directly, without rinse, to aniline blue solution and stained for 5–10 minutes. They were rinsed briefly in distilled water and differentiated in 1% acetic acid solution for 2–5 minutes.

They were washed in distilled water, dehydrated very quickly through 95% ethyl alcohol, absolute ethyl alcohol and cleared in xylene. They were mounted with resinous mounting medium [39].

### Immunohistochemical staining

Immunohistochemical staining was used to determine the MMP-9 and IL-17 expression around the grains of different mycetoma causatives agents. The deparaffinized tissue sections were mounted onto 3-aminopropyltriethoxysilane coated slides. Antigen retrieval was performed by incubating the sections in citrate buffer solution in water bath at 96°C for ten minutes as described previously [39].

After antigen retrieval, the tissue sections were rinsed first in distilled water and then with Tris buffer saline (TBS), followed by treatment with peroxidase block (3% hydrogen peroxide in methyl alcohol) for 15 min to quench endogenous peroxidase activity. The slides were then placed in a humid chamber. Then, the slides were drained and rinsed in two successive changes of Tris buffer (wash buffer), for five mins each. Non-specific protein-protein interactions were blocked by treating and incubating the tissue sections with the power block (casein in phosphate buffered saline) for 10 minutes in a humid chamber. The remaining solution was drained from the slides. For staining MMP-9, the sections were then incubated in the ready to use primary antibody (MMP-9); rabbit monoclonal antibody (class IgG, clone EP1255Y, BioGenex) at room temperature in the humid chamber for 30 mins. The remaining solution was drained from the slides and rinsed in two changes of Tris buffer saline (washing buffer) as mentioned earlier. Sections were then treated with a reagent for enhancing the staining for 30 min, followed by rinsing in two changes of Tris buffer. Sections were then incubated with multilink secondary antibody solution (EPOS) for 30 min, followed by rinsing with wash buffer and treated with HRP label (HRP-conjugated streptavidin) for 30 min. For staining IL-17, sections were incubated with primary antibody abcam ab79056 (IL-17; 1/100 in PBS +10% serum) for 14 hours at 4°C after which, the sections were washed in washing buffer for four times. A Biotin-conjugated goat anti-rabbit was used as the secondary antibody; the sections were incubated for ten mins, followed by rinsing in the washing buffer for four times. Then the sections were stained with Streptavidin-Peroxidase and incubated for 10 minutes at room temperature, followed by rinsing four times in buffer. To visualize the binding of the antibodies, sections were treated with DAB chromogen/substrate 3, 3-DAB chromogen/H2O2/substrate buffer solution (1 ml of DAB buffer mixed with 2 drops of DAB chromogen in a mixing vial and allowed to stand for about 10 min), covered with drops of chromogen DAB buffer solution, and allowed to stand in the humid chamber for 10 min. During this period, brown staining was visualised with varying intensity on different sections. Then, the slides were rinsed first in distilled water followed by running tap water. The sections were then counterstained with Mayer’s hematoxylin solution for 5 minutes, rinsed in running tap water, and dehydrated, cleared and mounted with dibutyl phthalate and xylene (DPX). Lymphoid tissue sections were used as positive controls while specimens that were treated as above except for the fact that the primary antibody was omitted and were used as negative controls.

### IL-17 and MMP-9 expression scoring system

To determine the IL-17 and MMP-9 roles in the mycetoma granuloma, a scoring system was designed to grade the expression. The tissue sections which didn’t express MMP-9 and IL-17 were considered negative. Positive tissue sections were further classified using a two-level scoring system; the quantity and intensity staining systems. The quantity staining corresponded to the percentage of immunoreactive cells and it is as follows, No staining = 0, 1–10% of cells = 1, 11–50% of cells = 2, 51–70% of cells = 3 and >70% = 4.

The intensity staining score was ranged as follows: No staining = 0, weak staining = 1, moderate staining = 2, and strong staining = 3.

Both the staining quantity and intensity were determined by the total Immunohistochemical (IHC) score. It ranged from 0 to 12, IHC score ranged between 0 to 4 were considered as low levels of expression while score from >4 to 12 were high levels of expression [40, 41].

The studied sections were interpreted by three independent expert pathologists. Photomicrographs were taken with Olympus SP-350 camera (Olympus Imaging America Inc., Center Valley, Pennsylvania, United States).

### Statistical analysis

The obtained data were processed using the Statistical Package for Social Sciences (SPSS version 11). Chi-square, ANOVA tests were used for data analysis to determine if IL-17A or MMP-9 expression was linked to causative agent, gender, lesion size or duration of the disease. To determine if IL-17A and MMP-9 expression was correlated Pearson correlation test was used. In general, p values less than 0.05 were considered statistically significant. All statistical analysis was performed using the statistical package SPSS11.0 (SPSS Incorporated, Chicago).

### Ethical statement

Study ethical clearance was obtained from Soba University Hospital Ethical Committee. Individual patient’s informed consent proved to be not necessary for this study as the surgical biopsies were part of routine patients’ management. All data anonymized before analyzed.

## Results

**Fig 1:**
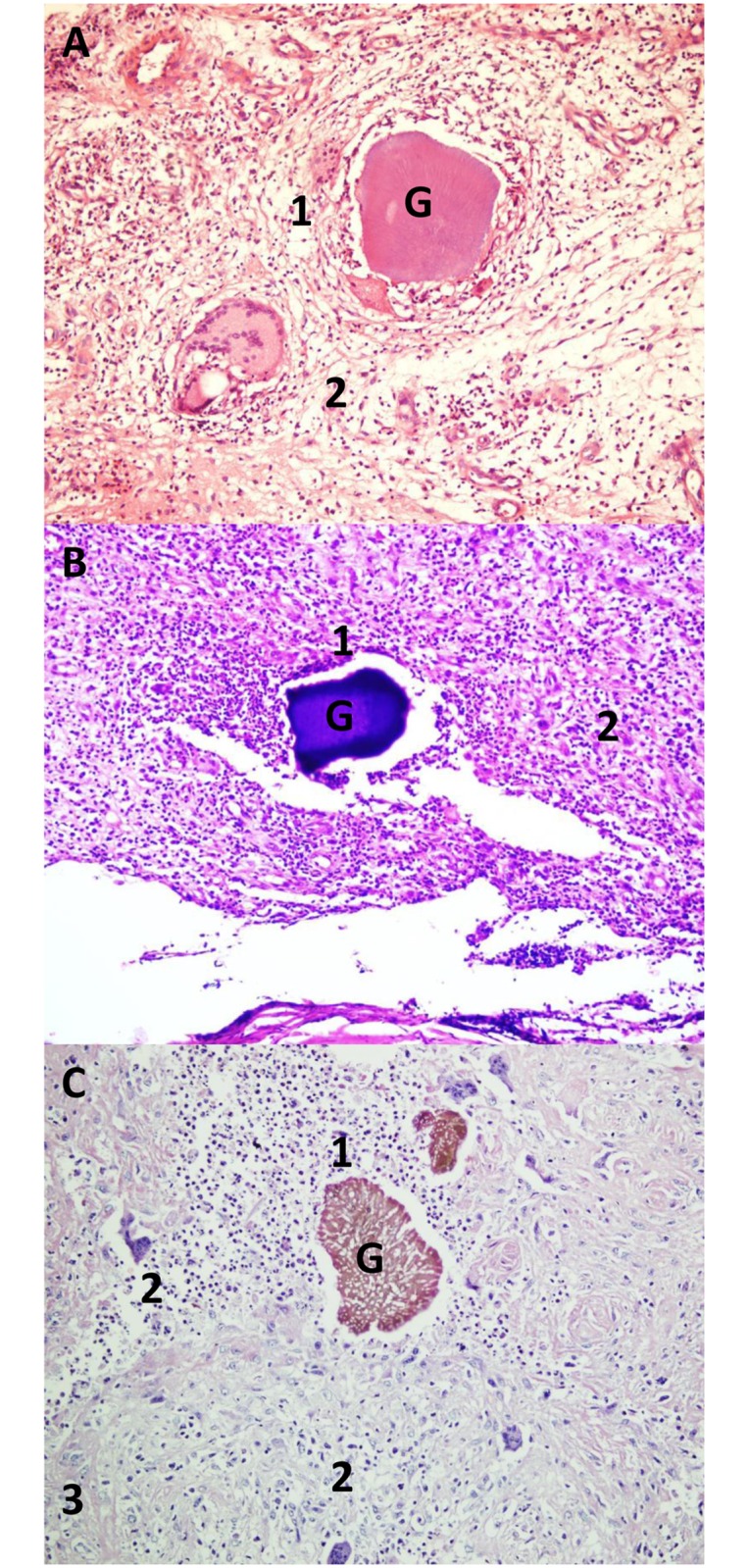
Hematoxylin and eosin stained sections demonstrating the grains (G) of A; *S*. *somaliensis*, *B; A*. *pelletieri and C; M*. *mycetomatis*, grains highlighted different inflammatory zones, Zone 1, 2 and 3 that surrounding the grains (G) (H and E, X 10). This figure is composed of representative pictures from single patients. Differences among individual patients were noted, however we chose pictures which correlated with the majority of each group of patients.

**Fig 2:**
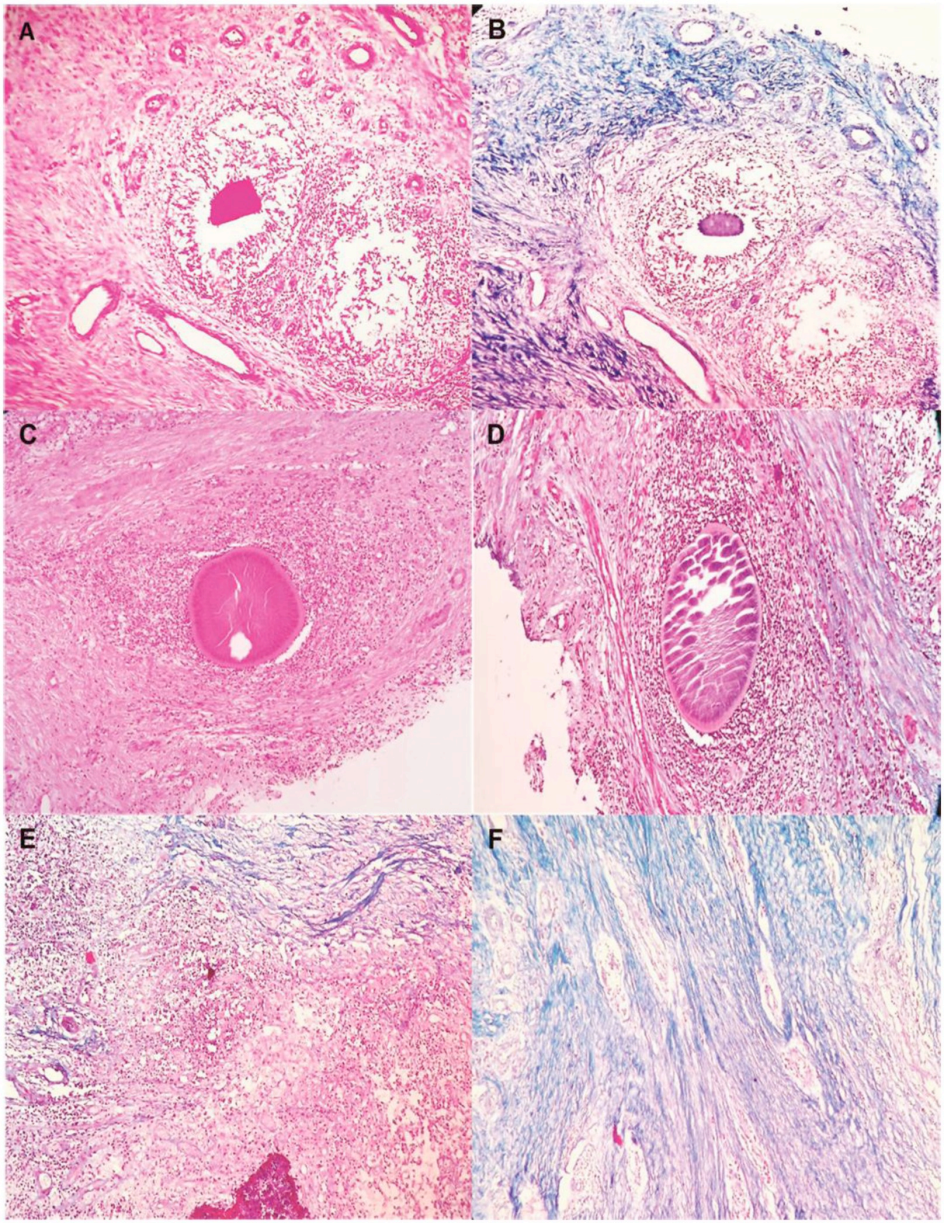
Collagen deposition around different mycetoma causatives agent; in this figure a representative picture of the *A*. *pelletieri* grain insight the subcutaneous tissue from one particular patient is shown. In panel A, a HE staining is performed. In panel B, a Masson trichrome staining of the same area is shown. As is seen on this slide, collagen (coloured blue) is mainly seen within zone 2 as a fine thin bundles that surrounded the grains. In panel C, a HE staining is performed from patient with *S*. *somaliensis;* and D, collagen fibers is mainly seen within zone 2 as a fine thin bundles that surrounded the grains; in Panels E and F showed a collagen fibers in *M*. *mycetomatis*, with thick collagen bundles. This figure is composed of representative pictures from single patients. Differences among individual patients were noted, however we chose pictures which correlated with the majority of each group of patients.

The current study included 100 patients with confirmed mycetoma. All patients were on treatment. There were eighty-three males and 17 females; their ages ranged between seven and 61 years with a mean age of 26.9± 10.9 years. The disease duration ranged between one and 12 years with a mean duration of 3.7± 2.8 years. The lesion sizes were small (<5cm) (14%), moderate (5-10cm) (62%), and massive (24%) (>10cm). The most common site of the infection was the foot (56%), right ankle (19%), hand (12%), leg (7%) and the least common site was the big toe (6%). (Table 1). Out of these 100 patients, 80 patients had mycetoma caused by *M*. *mycetomatis*, 12 patients had mycetoma caused by *S*. *somalienisis* and 8 patients had mycetoma caused by *A*. *pelletieri* (Table 1). As can be seen in Fig 1, for all three species the inflammation surrounding the mycetoma grain was composed of three zones as described in literature (Fig 1). Furthermore, as can be seen in the Masson Trichrome stained sections, both eumycetoma and actinomycetoma grains were encapsulated by collagen fibres. For eumycetoma these collagen fibers usually presented as thin rings more closely to the grain and were then followed by dense collagen bundles at the periphery of the ring. This differed from the collagen fibres seen in actinomycetoma lesions. In these lesions, the bacterial grain was usually only surrounded by thin collagen fibers, no dense collagen bundles were noted. (Fig 2).

**Table 1:** The clinical characteristics of the studied population.

Characteristics	No. (%)
Gender	
Male	83%
Female	17%
Lesion Size	
Small (Less than 5 cm)	14%
Moderate (5–10 cm)	62%
Massive (more than 10 cm)	24%
Site	
Foot	56%
Right ankle	19%
Hand	12%
Leg	7%
Big toe	6%

### IL-17A is mainly expressed in zones I and II of large mycetoma lesions

**Fig 3:**
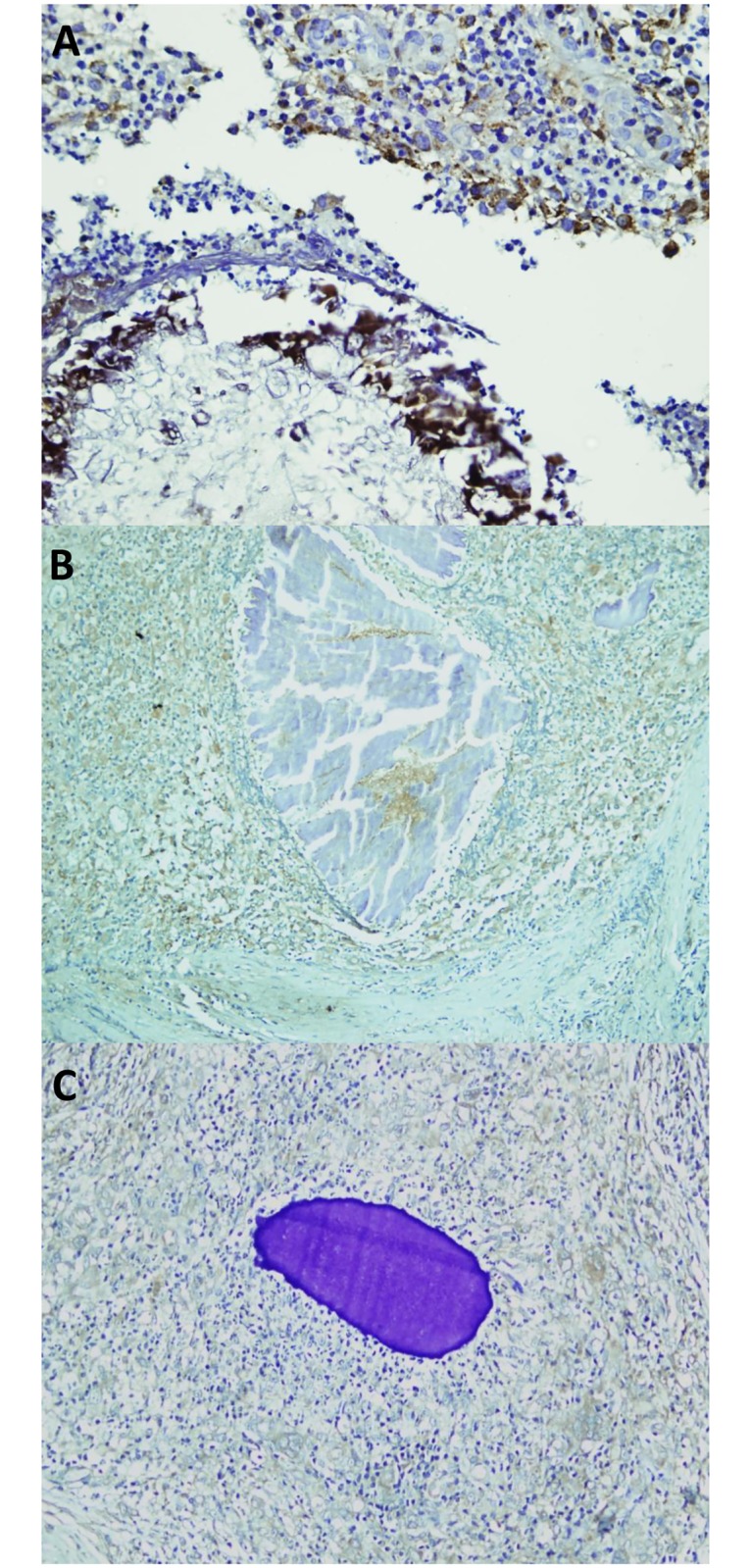
IL-17A expression in *M*. *mycetomatis* (A), *S*. *somaliensis* (B) and *A*. *pelletieri* (C) lesions. IL-17A staining was seen as a brown cytoplasmic staining in cells. This figure is composed of representative pictures from single patients. Differences among individual patients were noted, however we chose pictures which correlated with the majority of each group of patients.

As can be seen in Fig 3, IL-17A was expressed in the cells surrounding the grains. Since the inflammatory reaction surrounding the mycetoma grains differs per zone it was also assessed if the IL-17A expression differed per inflammation zone. For all three causative agents, the highest expression was noted in zones I and II, lower expression was noted in zone III (Fig 3). IL-17A expression was noticed within the cytoplasm of neutrophils, macrophages and Lymphocyte cells. Strikingly, slides in which a high expression of IL-17A was seen, angiogenesis were more prevalent than in slides were this was not the case. To assess if different IL-17A expression patterns were seen with the different causative agents, our results showed that a significant difference in IL-17A expression between the different causative agents was seen (Kruskal-Wallis Test, p = 0.009). No differences in IL-17A expression were noted between males and females. However, a significant higher expression was found in large lesions compared to small lesions (p = 0.012), or in lesions with a longer disease duration (p = 0.020) (Table 2).

**Table 2:** Correlations between the IL-17 expression and the patient’s clinical data.

Characteristics		IL 17 expression			Total	*p* value
		Negative	Low expression	Highly expressed		
Gender	Male	11	26	46	83	0.765
	Female	2	4	11	17	
Lesion Size	Less than 5 cm	2	3	10	15	0.012
	5–10 cm	11	23	29	63	
	More than 10 cm	0	4	18	22	
Duration	Less than 3 years	3	10	28	41	0.020
	3 to 6 years	10	20	24	54	
	More than 6 years	0	0	5	5	

### MMP-9 is mainly expressed in zones II and III surrounding the mycetoma grain

**Fig 4:**
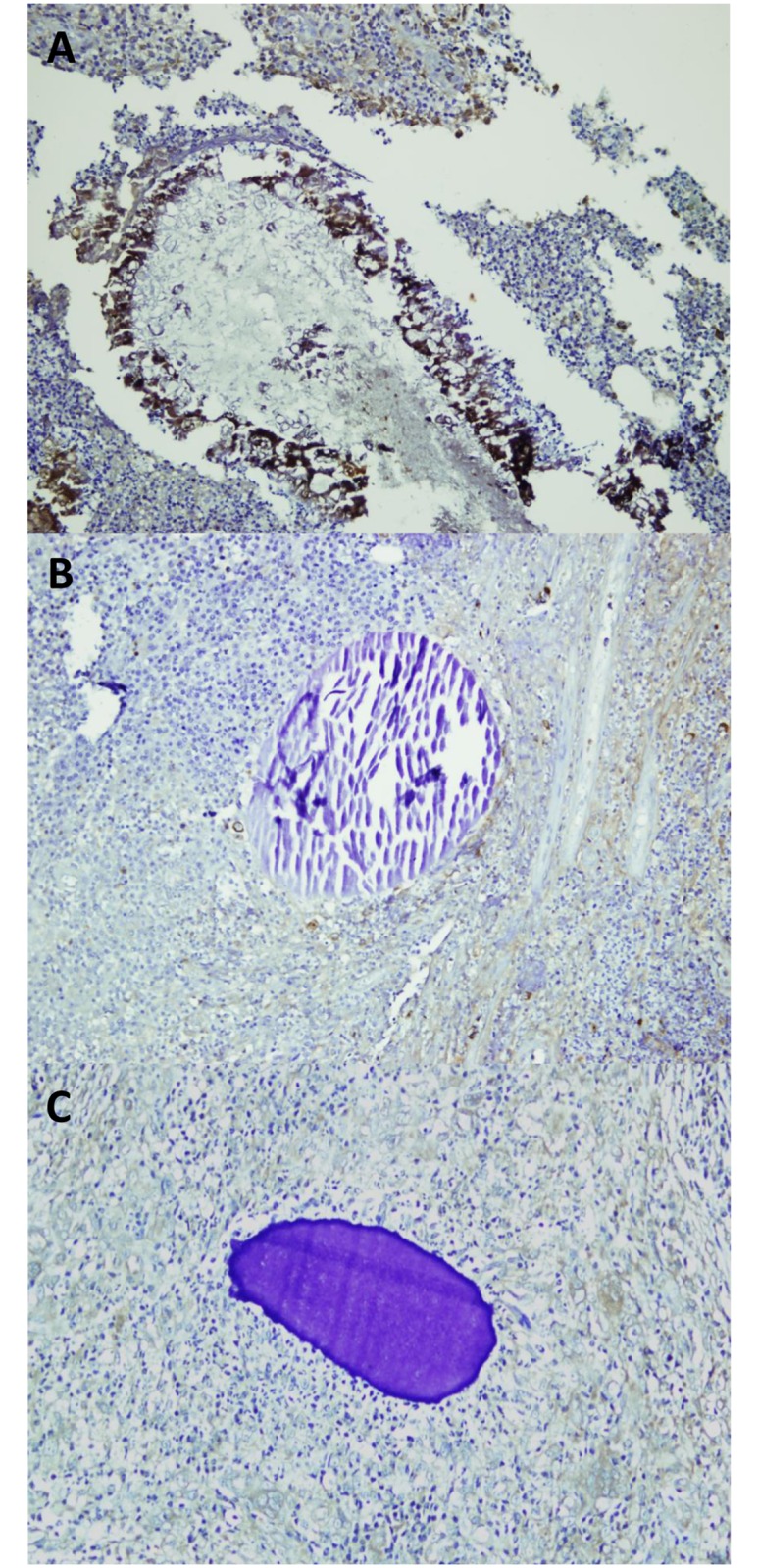
MMP-9 expression in *M*. *mycetomatis* (A), *S*. *somaliensis* (B) and *A*. *pelletieri* (C). Stromal expression (i.e. connective tissue parenchymal) of MMP-9 was negative in all the areas which contained thick bundles of collagen fibers (arrow head) (X 10). This figure is composed of representative pictures from single patients. Differences among individual patients were noted, however we chose pictures which correlated with the majority of each group of patients.

**Fig 5:**
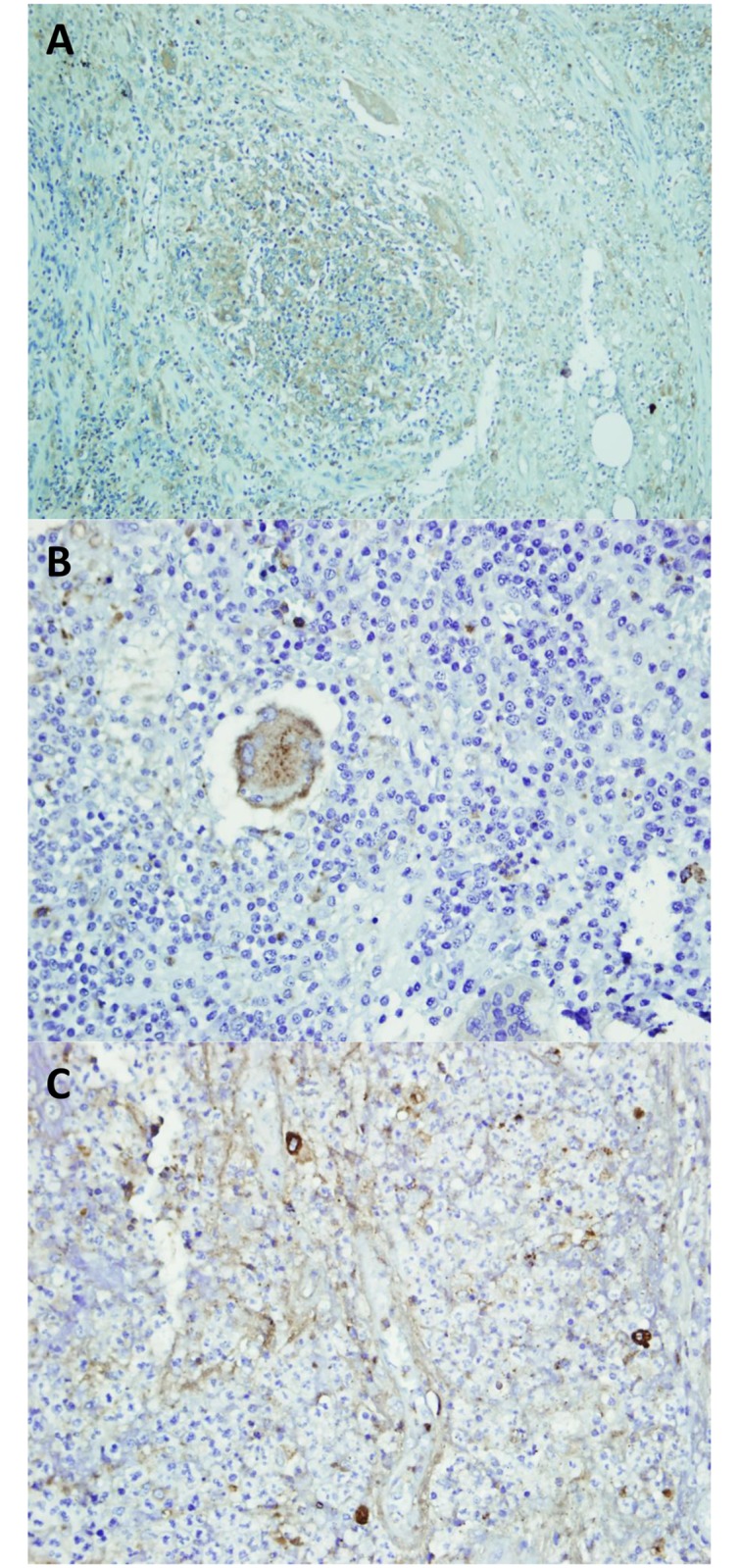
**Showed the expression of IL-17 within tissue sections; A: IL- 17 was expressed in T-lymphocyte cells and neutrophils**. B; in giant cells, C; Stromal cells were noticed to expressed the IL 17. This figure is composed of representative pictures from single patients. Differences among individual patients were noted, however we chose pictures which correlated with the majority of each group of patients.

MMP-9 expression was detected as brown cytoplasmic stain within the cells. It was found to be mainly expressed in Zones II and III surrounding the mycetoma grain (Fig 4). Only limited expression was found in Zone I. Stromal expression (i.e. connective tissue parenchymal) of MMP-9 was negative in all the areas which contained thick bundles of collagen fibers. As can be seen in Fig 5, MMP-9 was more highly expressed in *A*. *pelletierii* and *S*. *somaliensis* biopsies compared to *M*. *mycetomatis* (Kruskal-Wallis Test, p < 0.0001). No difference in MMP-9 expression was noted between males and females. However, a significant higher MMP-9 expression was found in lesions with a longer disease duration (p < 0.0001), on other hand no significant higher MMP-9 expression was found in large lesions compared to small lesions (Table 3).

**Table 3:** Correlations between the MMP-9 expression and the patient’s clinical data.

Characteristics		MMP 9 expression			Total	*p* value
		Negative	Low expression	Highly expressed		
Gender	Male	7	60	16	83	0.192
	Female	4	9	4	17	
Lesion Size	Less than 5 cm	3	8	4	15	0.053
	5–10 cm	8	46	9	63	
	More than 10 cm	0	15	7	22	
Duration	Less than 3 years	7	22	12	41	<0.0001
	3 to 6 years	4	47	3	54	
	More than 6 years	0	0	5	5	

### Correlation between IL-17A expression and MMP-9 expression and collagen deposition

**Fig 6:**
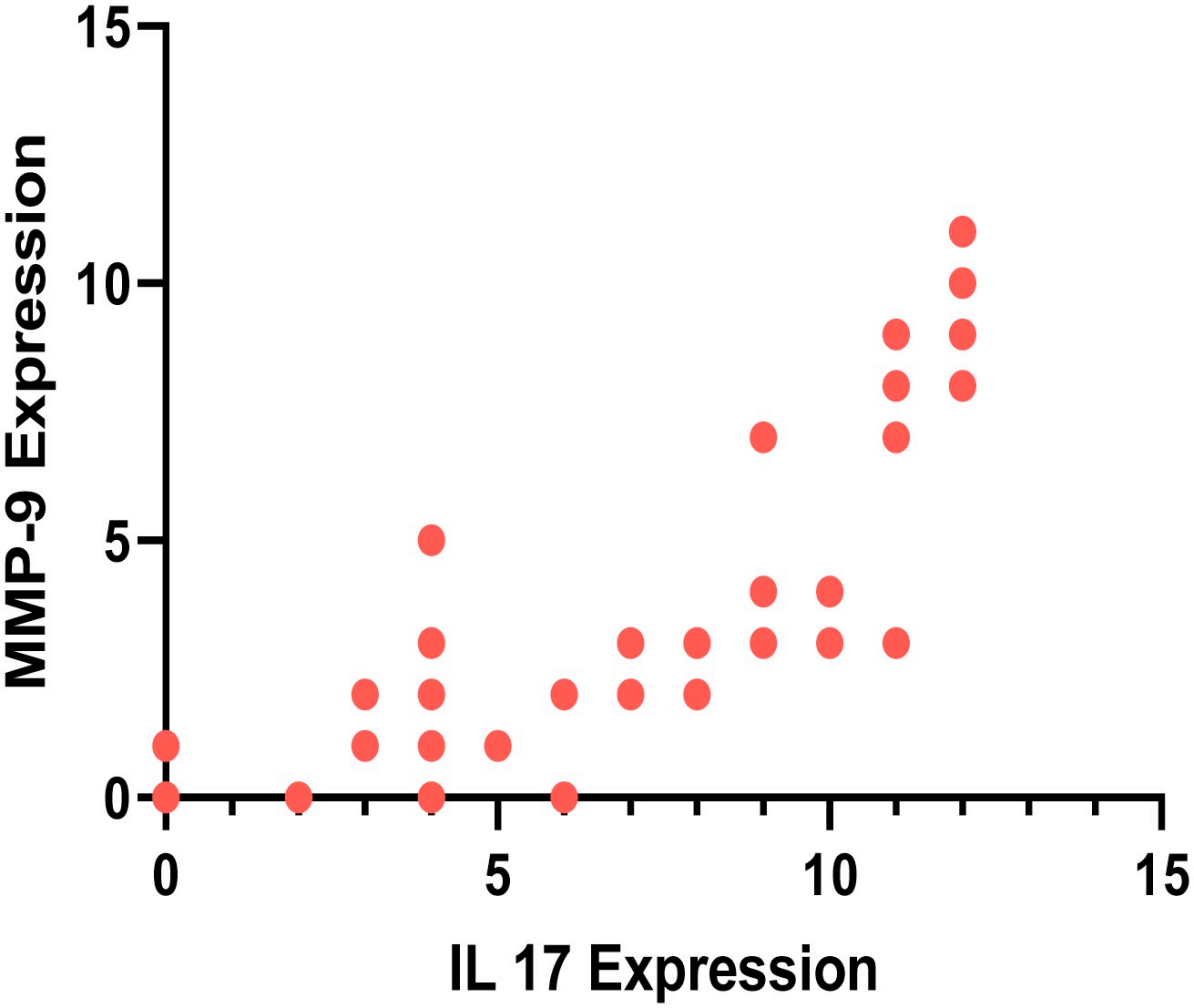
Correlation between IL-17A expression intensity and MMP-9 expression intensity, r = 0.8887, p value <0.0001.

To determine if there was a correlation between IL-17A expression and MMP-9 expression the expression levels for each of the samples were plotted. As can be seen in Fig 6, there was a correlation between IL-17A expression and MMP-9 expression (p < 0.0001).

## Discussion

The immunopathological mechanisms involved in the chronic granulomatous inflammation reaction in mycetoma are not well understood but evolving body of evidence suggested that cytokines play a critical role in granuloma formations. Both Th1 and Th2 cytokines were observed to be expressed in the mycetoma granuloma and the Th2 cytokines appeared to be more dominant expressed [15, 19]. In the current study we demonstrated that also the Th17 signature cytokine, IL-17A was expressed in the mycetoma granuloma of different mycetoma causative agents including *M*. *mycetomatis*, *S*. *somaliensis* and *A*. *pelletieri*. In eumycetoma caused by *M*. *mycetomatis* it was found to be mainly expressed in zones I and II, lower expression was noted in zone III. A similar expression pattern was found for actinomycetoma grains caused by *S*. *somaliensis* and *A*. *pelletierii*. To find IL-17A expressed in polymorphonuclear cells surrounding fungal or actinomycete infections is not unique and it was also described in granulomas in paracoccidioidomycosis [42], and in *Mycobacterium bovis* infections [43] and even in mycetoma lesions caused by *Nocardia brasiliensis* [21], and *Phialophora richardsiae* [44]. In the *M*. *bovis* infection model it was demonstrated that IL-17A deficient mice showed impaired granuloma formation, indicating the importance of IL-17A in establishing granuloma’s around actinomycete infections [45]. However for *M*. *bovis* IL-17A was mainly expressed in the early stages of the disease [43]. Also in experimental *Nocardia brasiliensis* mycetoma, IL-17A was found to be increased mainly at the beginning of the infection [21], at the time the *N*. *brasiliensis* mycetoma granuloma is formed. As with *M*. *mycetomatis*, *A*. *pelletieri* and *S*. *somaliensis* mycetoma, the *N*. *brasiliensis* granuloma is characterized by neutrophils and the high expression of IL-17A in the beginning of the infection could be responsible for the recruitment of neutrophils [21].

One of the shortcommings of our study is that we were able to look at expression at a fixed moment of time, a time point in which the mycetoma granuloma was already formed. With immunohistochemistry we therefore were only able to demonstrate the presence of IL-17A and MMP-9, not the time point when they were formed. It is therefore impossible to determine the precise function of IL-17A and MMp-9 in the formation of the mycetoma granuloma, we could only determine their presence. However, taken this in mind, we did found differences in the expression level of IL-17A in the different lesions. The highest IL-17A expression was found in the largest lesions and the lesions with the longest duration. One explanation could be that these might also be the most aggressive lesions and that the continues expression of IL-17A results in the massive lesions. Indeed, in gastric cancer higher IL-17A expression was also found in more aggressive and larger tumours [46]. Continues IL-17A expression, will result in a continues influx of neutrophils at the site of infection and a continuous influx of tissue damage and remodeling due to the production of ROS and metalloproteases. However, since in our study we were only to look at a single time point per patient, it is impossible to say if IL-17A is continuously expressed and how it exactly interacts with other cytokines and chemokines within mycetoma lesions. Further studies are needed to investigate the role of the different cytokines and their interplay in more detail.

If indeed tissue damage plays an important role in the formation of the mycetoma granuloma a role of IL-17A could be proposed. IL-17A is known to up-regulate MMP-9 expression, and to increase the level of active MMP-9 [47–49]. In our study we also demonstrate the positive correlation between IL-17A and MMP-9 expression.

However, the presence of any MMP at the site of infection does not necessarily mean that the MMP is activated. Geneugelijk and colleagues demonstrated the presence of both MMP-2 and MMP-9 in eumycetoma lesions caused by *M*. *mycetomatis*. Although MMP-2 and MMP-9 were both found to be expressed at the site of infection, only in 36% of patients with *M*. *mycetomatis* the activated form of MMP-9 was present. The activated form of MMP-2 was completely absent [16]. Unlike IL-17A expression, we did found differences in MMP-9 expression between *M*. *mycetomatis* and the actinomycetoma lesions *A*. *pelletierii* and *S*. *somaliensis*. MMP-9 was expressed in larger quantities in *A*. *pelletierii* and *S*. *somaliensis* lesions compared to *M*. *mycetomatis*. This correlated also with the smaller collagen capsules encountered in *A*. *pelletieri* and *S*. *somaliensis* compared with *M*. *mycetomatis*. The difference could also be due to the differences in treatment. All *M*. *mycetomatis* patients were on itraconazole treatment at the time the biopsy was performed. In a paraccocidiomycosis mouse model it was demonstrated that itraconazole treatment resulted in a significant reduction of MMP-1α, MMP-8 and MMP-13 expression, while an increase in expression was noted for MMP-12 and MMP-14 [49]. MMP-9 was not measured in that study; however itraconazole treatment could have also influenced the expression of MMP-9. Furthermore, in a study conducted by Yuan and his associates they investigated the expression of MMPs notably MMP-8, -9, -10, -12, -13, -1 and tissue inhibitors of metalloproteinases (TIMPs) during the inception and progression of experimental keratomycosis due to *Candida albicans* using BALB/c mice. Their results showed that Mock-infected corneas demonstrated moderate epithelial staining and minor stromal staining for MMP-8, -9, -13, and TIMP-1 while corneas from infected animals had increased staining for these proteins throughout the epithelium and stroma [50].

In summary, the results obtained in the present study demonstrated that IL-17A and MMP-9 were expressed in the granuloma of different mycetoma causative agents and that the expression of IL-17A was more extensively in larger leasions and lesions with a longer disease duration. We also deomonstrated a positive correlation between IL-17A expression levels and MMP-9 expression levels. However, since we only demonstrated expression, more studies are needed to determine the roles of IL-17A and MMP-9 in the granuloma formation and to provide further insights in the mycetoma immunopathogenesis. The observations presented here are the first step and indicate tha IL-17A and MMP-9 are present within the grain. The functions of IL-17A and MMP-9 in the formation of the grain will be studied in future studies.

## Supporting information

S1 Checklist STROBE checklist. (DOCX)
